# Synthesis and biophysical characterization of bioengineered cytochrome nanowires

**DOI:** 10.1007/s00249-026-01848-1

**Published:** 2026-05-23

**Authors:** M. Raquel Pacheco, Ana J. Carvalho, Marta A. Silva, Tomás Calmeiro, Nykola C. Jones, Søren V. Hoffmann, Leonor Morgado, M. Manuela A. Pereira, Elvira Fortunato, Carlos A. Salgueiro, Pedro Tavares, Alice S. Pereira

**Affiliations:** 1 UCIBIO − Applied Molecular Biosciences Unit, Department of Chemistry, NOVA School of Science and Technology, Universidade NOVA de Lisboa, 2829-516 Caparica, Portugal; 2 Associate Laboratory i4HB − Institute for Health and Bioeconomy, NOVA School of Science and Technology, Universidade NOVA de Lisboa, 2829-516 Caparica, Portugal; 3 CENIMAT/i3N, Department of Material Sciences, NOVA School of Science and Technology, Universidade NOVA de Lisboa, 2829-516 Caparica, Portugal; 4 ISA, Department of Physics and Astronomy, Aarhus University, DK-8000 Aarhus C, Denmark; 5 LAQV, REQUIMTE, Department of Chemistry, NOVA School of Science and Technology Universidade NOVA de Lisboa, 2829-516 Caparica, Portugal

**Keywords:** *Geobacter*, Multiheme *c*-type cytochromes, Cytochrome *c* PpcA, Protein nanowires, Thiol-ene coupling, Synchrotron radiation circular dichroism

## Abstract

*Geobacter* bacteria produce multiheme *c*-type cytochrome nanowires that are involved in long-range extracellular electron transfer. The ability of these protein nanowires to conduct electrical current makes them promising candidates for electronic devices, offering several functional and sustainable advantages over traditional materials. Therefore, this study focused on synthesizing hybrid protein fibers that mimic the natural *Geobacter* extracellular nanowires. To achieve this, a mutated PpcA triheme protein variant was used as the building block, with thiol-ene coupling employed to bind the protein molecules. This engineered PpcA variant (PpcAK9CK22C) maintained a structure similar to that of the native protein. Thermal denaturation studies revealed a two-state process, with a melting temperature of 62 ± 1 °C and an enthalpy change of 61 ± 2 kcal/mol. The new protein nanowires showed a lower heme group content than the precursor protein and displayed distinct secondary structure features, with a slight reduction in helical content and an increase in β-sheet and unordered structures. Their thermal stability also differed, as it could not be described by the same model applied to the PpcA variant. Despite these differences, the nanowires retained their ability to undergo redox cycling. Morphologically, they consisted of linear single-protein filaments extending over 300 nm in length.

## Introduction

*Geobacter* are gram-negative rod-shaped bacteria found in diverse environments, like terrestrial soil, aquifers, and aquatic sediments (Lovley et al. 2011; Reguera and Kashefi 2019). These bacteria are capable of using a broad range of compounds as both energy and carbon sources. Depending on the strain, they metabolize various organic acids, short-chain fatty acids, alcohols, and monoaromatic compounds (Lovley et al. 2011; Reguera and Kashefi 2019). For electron acceptors, *Geobacter* can utilize ferric compounds, Mn(IV) oxides, U(VI), Ag(I), elemental sulfur, chlorinated ethenes, humic acids and analogs, nitrate, fumarate, malate, or molecular oxygen (Law et al. 2008; Lovley et al. 2011; Reguera and Kashefi 2019; Salgueiro et al. 2022).

The remarkable ability to utilize such a wide variety of compounds for cellular respiration is achieved, in part, by expressing a vast diversity of *c*-type cytochrome proteins, which possess one or more heme groups covalently bound to the peptide chain. *Geobacter* bacteria can express a striking number of different *c*-type cytochrome proteins, ranging from 61 to 104 (Butler et al. 2010). A significant majority of these, between 75% and 89%, are multiheme *c*-type cytochromes (Butler et al. 2010). In the known structures of these proteins, iron ions are generally axially coordinated by two histidine residues. The heme groups are typically stacked or arranged in a T-shape, with adjacent groups positioned no more than 16 Å apart, enabling efficient and rapid electronic transfer (Salgueiro and Dantas 2016; Salgueiro et al. 2022). The presence of multiple heme groups expands the overall working redox potential range of the protein. This functional versatility arises from the combined reduction potential of each heme and the influence of heme-heme interactions (Salgueiro and Dantas 2016; Salgueiro et al. 2022). The existence of several heme groups also allows for cooperative electron transfer, depending on the properties of the neighboring hemes (redox interactions) and the redox-Bohr centers (Salgueiro and Dantas 2016; Salgueiro et al. 2022).

The *c*-type cytochromes are found in distinct subcellular locations. Some are associated with the inner membrane, such as ImcH (Levar et al. 2014) and CbcL (Zacharoff et al. 2016). Others reside in the periplasm, such as the PpcA-E protein family (Morgado et al. 2010). Others are in contact with the outer membrane, including OmcE, OmcS, or OmcT (Mehta et al. 2005), where they can form complexes with other proteins, like OmcB or OmcC (Liu et al. 2014b).

In addition to carrying electrons during cellular respiration, cytochrome *c* proteins perform several other crucial functions. They can perform catalytic activities (Hoffmann et al. 2009; Seidel et al. 2012; Aklujkar et al. 2013; Campeciño et al. 2020) and mediate direct interspecies electron transfer (Reguera and Kashefi 2019). Furthermore, these proteins can act as cellular capacitors to store respiratory electrons (Esteve-Núñez et al. 2008; Malvankar et al. 2012) or they can assemble into cytochrome-based nanowires.

Cytochrome-based nanowires constitute approximately 10% of the filaments emanating from *Geobacter sulfurreducens* cells (Liu et al. 2021) and are also believed to be involved in long-range extracellular electron transfer (Filman et al. 2019; Wang et al. 2019). These nanowires can comprise hundreds of proteins, reaching micrometers in length (Wang et al. 2019).

There are three known types of cytochrome nanowires. The first type is formed by the OmcS hexaheme cytochrome *c* protein (Filman et al. 2019; Wang et al. 2019). Its six heme groups are arranged in parallel-stacked pairs, which are perpendicular to each other along the length of the protein chain, and most of them are completely shielded from the solvent. The polymerization of these nanowires occurs when His-16 from one monomer coordinates the iron atom of heme V in the adjacent monomer. The polymerization process also involves forming a heme group pair with one cofactor from each monomer. These nanowires form a 1-start left-handed helix with a thickness of ~ 4 nm, ~ 4.3 monomers per turn, and a pitch of ~ 200 Å. Each monomer contributes with a rise of about 47 Å and a rotation of approximately − 83°. These nanowires also have a direct current conductivity of approximately 35 mS/cm.

The second type of cytochrome nanowires is comprised of a tetraheme protein, OmcE (Wang et al. 2022). As in the previous type, OmcE nanowires are filaments with a repeating pattern and a conserved heme packing arrangement. They are 4 nm-wide filaments, 1-start helices with a pitch of 207 Å and 6.1 subunits per turn. Each subunit originates a rise of ~ 34 Å and a twist of ~ 59°. OmcE protein suffers post-translational modifications and these filaments are highly glycosylated.

The last group is composed of the octaheme cytochrome OmcZ_S_ (Yalcin et al. 2020). This type of nanowires has a diameter of ~ 2.5 nm and, as helical parameters, a rise of 57 Å and a rotation of 160°. Additionally, it presents a conductivity of 30 S/cm at pH 7.

Electrically conductive protein nanowires show great promise for use in electronic devices, as, for example, sensors (Marques et al. 2015), offering several functional and sustainable advantages over traditional materials. Among these benefits are their unlimited production at lower energy costs, the possibility of modulating their properties, and their biodegradability (Lovley and Holmes 2020; Lovley and Yao 2021). The in vitro synthesis of cytochrome nanowires could overcome challenges associated with their in vivo production, while also simplifying the purification process (Lovley and Yao 2021). Several approaches have been attempted to create electrically conductive protein nanowires, but none have successfully replicated the cytochrome nanowires produced by *Geobacter*. One approach used the filamentous protein γ-prefoldin as a scaffold, which was conjugated with either one or two molecules of the tetraheme cytochrome 𝑐_3_ via SpyTag-SpyCatcher domains (Chen et al. 2020). Another method involved producing rectangular crystals from a small tetraheme cytochrome, resulting in structures with a cross-section of ~ 100 proteins (~ 30 nm) (Huang et al. 2020). Researchers also synthesized filaments and two-dimensional networks through heme-heme pocket interactions from the monoheme cytochrome 𝑏_562_ functionalized with a heme moiety on its surface (Kitagishi et al. 2007, 2009; Oohora et al. 2017). Furthermore, researchers developed amyloid fibril scaffolds derived from the Src homology 3 domain, which displayed functional cytochromes 𝑏_562_ on their surface (Baldwin et al. 2006; Forman et al. 2012).

In the present work, we aimed to create protein polymers that mimic the extracellular nanowires found in *Geobacter* bacteria. We chose *Geobacter sulfurreducens* PpcA cytochrome as the building block because it is a very well-characterized protein (Pessanha et al. 2006; Fernandes et al. 2008; Morgado et al. 2008, 2017). It is also structurally simpler than OmcS, OmcE, or OmcZ_S_, as it only possesses three heme cofactors. To covalently bind the PpcA molecules, we followed the same synthetic approach previously utilized to produce the mini-ferritin (DpsT10C) protein fibers (Pacheco et al. 2020). Therefore, to enable the thiol-ene coupling click chemistry reaction, the PpcA protein was engineered to contain two cysteine residues at approximately diametrically opposed positions.

## Methods

### Materials

All commercially available reagents and solvents were of pro-analysis grade and were used as acquired without further purification. 1,2-bis(allyloxy)ethane was synthesized based on the procedure described by Abramov et al. (2002).

### Preparation of the PpcAK9CK22C protein variant

The PpcAK9CK22C protein variant (with lysine residues at positions 9 and 22 replaced by cysteine) was produced by site-directed mutagenesis using the *Geobacter sulfurreducens* PpcA gene cloned into the pCK32 periplasmic expression vector as template (Londer et al. 2002). The desired mutations were introduced sequentially with the NZYMutagenesis kit (NZYtech, Lda., Portugal) following the manufacturer’s recommendations. Oligonucleotide primers, forward (K9C_f: 5’-C GAC ATC GTC CTC AAG GCC TGT AAC GGT GAT GTG AAG TTC CC-3’, K22C_f: 5’-CG CAC AAG GCC CAC CAG TGT GCT GTT CCC GAC TGT AAG AAG TGC-3’) and reverse (K9C_r: 5’-GG GAA CTT CAC ATC ACC GTT ACA GGC CTT GAG GAC GAT GTC G-3’, K22C_r: 5’-GCA CTT CTT ACA GTC GGG AAC AGC ACA CTG GTG GGC CTT GTG CG-3’), were designed and analyzed for optimization with the Thermo Scientific™ web tool T_m_ Calculator for Primers (Thermo Fisher Scientific Inc, USA). The primers were synthesized and purified by Invitrogen (USA). Primers were annealed at 60 °C and extended at 68 °C. After digesting the PCR product with DpnI to remove the parental plasmid DNA, the enzyme was inactivated at 80 °C for 20 min. The resulting mutated plasmid DNA was then utilized to transform *Escherichia coli* DH5α competent cells. Finally, clones were analyzed by DNA sequencing of pCK32-PpcAK9CK22C plasmid DNA (STABVIDA, Lda., Portugal).

### Production of PpcA proteins

Overexpression and purification of wild-type PpcA and PpcAK9CK22C proteins were performed using the same approach previously reported for PpcA (Londer et al. 2002; Pokkuluri et al. 2004; Morgado et al. 2008). *Escherichia coli* BL21(DE3) cells harboring the pEC86 plasmid (which encodes the cytochrome *c* maturation gene cluster *ccmABCDEFH*) were transformed with the plasmid containing the gene of interest. For the inoculum (2 × 50 mL), the cells were grown overnight, at 30 °C, in 2xYT medium containing 34 µg/mL of chloramphenicol and 100 µg/mL of ampicillin. Six 1 L media were inoculated with 10 mL of the inoculum, and the cultures were grown to an optical density at 600 nm of ~ 1.5. Protein expression was then induced with 10 µM of isopropyl β-d-1-thiogalactopyranoside and allowed to proceed overnight, at 30 °C. After pelleting (6,400 *g*, for 15 min at 8 °C), the cells were resuspended in lysis buffer (30 mL of 100 mM Tris-HCl, pH 8.0, 0.5 mM ethylenediaminetetraacetic acid, 20% sucrose, 0.5 mg/mL lysozyme per liter of culture) and incubated for 15 min at room temperature. An equal volume of cold water was added, and the cell suspension was stirred on ice for 15 min. The periplasmic fraction was collected by centrifugation at 14,700 *g* for 20 min at 8 °C. The supernatant was first dialyzed at 4 °C for 5 h against 10 mM Tris-HCl, pH 8.5. The buffer was then replaced with a fresh solution, and dialysis was continued overnight. After dialysis, the periplasmic fraction was centrifuged at 45,000 *g* for 1 h. For the PpcAK9CK22C protein, all dialysis and purification buffers contained 1 mM dithiothreitol (DTT).

The proteins were first purified using cationic ion exchange chromatography with two Bio-Scale™ Mini UNOsphere™ S cartridges (5 mL each, Bio-Rad, USA) connected in tandem. The adsorbed proteins were eluted by applying a linear gradient from 0 mM to 300 mM NaCl in 10 mM Tris-HCl, pH 8.5, at 1.5 mL/min (total volume of 150 mL). The final purification was performed using size exclusion chromatography with a Superdex™ 75 prep grade column (XK 16/70, 120 mL, GE Healthcare, currently Cytiva, USA) equilibrated with 100 mM sodium phosphate buffer, pH 8.0, at a flow rate of 0.5 mL/min. Both chromatographic steps were performed utilizing an ÄKTA system (ÄKTAprime plus in the first step and ÄKTA pure™ in the second, GE Healthcare, currently Cytiva, USA) and fractions of 1 mL were collected. After each chromatographic step, protein purity was evaluated by SDS-PAGE using 15% polyacrylamide gels under reducing conditions with β-mercaptoethanol. The NZYColour Protein Marker I served as molecular mass reference (NZYtech, Lda., Portugal). The protein bands in the polyacrylamide gels were stained with BlueSafe (NZYtech, Lda., Portugal). Based on their purity, the fractions were pooled and concentrated in an Amicon^®^ Ultra-15 3 kDa centrifugal filter device (Millipore^®^, Germany). The final protein fractions were analyzed using both UV-Visible spectroscopy and Tricine-SDS-PAGE (12.5% polyacrylamide) (Schägger and von Jagow 1987) as described below for the cytochrome nanowires. Protein concentration was determined by measuring the absorbance of the reduced protein at 552 nm, using a Thermo Scientific™ Evolution™ 201 UV-Visible spectrophotometer (Thermo Fisher Scientific Inc, USA). A molar extinction coefficient of 32.5 mM^−1^cm^− 1^ per heme group was assumed for both proteins. The heme content of the protein was calculated with the Abs_408 nm_/Abs_210 nm_ ratio from the oxidized spectra.

### Preparation of PpcAK9CK22C for the coupling reaction

Immediately before the click chemistry coupling reaction, the PpcAK9CK22C protein was incubated with 10 mM DTT at room temperature for 30 min. The DTT was removed utilizing a PD SpinTrap™ G-25 column (Cytiva, USA), eluting the protein with 50 mM potassium phosphate buffer (KPB), pH 5.7, 50 mM NaCl. The concentration of the protein was determined as described in the previous section.

### Production of cytochrome nanowires by PpcAK9CK22C protein coupling

The synthesis of PpcA-based nanowires was carried out by adapting the protocol previously used for producing the long Dps protein polymers (Pacheco et al. 2020).

A UV-LED spotlight source Lightningcure^®^ LC-L1V5, equipped with a LED head unit of standard type (Hamamatsu Photonics, Japan), was used to irradiate the protein samples at 365 nm. The light, focused by a condenser lens to a 6 mm diameter spot, was positioned 1.5 cm from the clear side of a 1.4 mL quartz cuvette (1 cm optical pathlength, Spectrosil^®^ quartz, Starna Scientific Ldt., UK). Irradiation was performed at 50% intensity under stirring at room temperature.

A 294.6 µL reaction mixture was prepared containing 50.9 µM PpcAK9CK22C protein and 50.8 µM 1,2-bis(allyloxy)ethane in 50 mM KPB, pH 5.7, 50 mM NaCl, 0.07% dimethylformamide (v/v). The photoinitiator lithium phenyl-2,4,6-trimethylbenzoylphosphinate (LAP) was added in three separate aliquots of 1.8 µL from a 5.6 mM stock solution (prepared in 50 mM KPB, pH 5.7, 50 mM NaCl) at 0 min, 30 min, and 60 min of irradiation. The irradiation process lasted a total of 120 min, after which the reaction mixture was frozen at − 20 °C to stop the reaction. The progression of the reaction was monitored by Tricine-SDS-PAGE, using 10 µL aliquots collected at 30-, 60-, and 120-min time points.

The coupling reaction product was washed with an equal volume of 50 mM KPB, pH 5.7, 50 mM NaCl in a Vivaspin^®^ 500 5 kDa centrifugal filter unit (Sartorius Lab Instruments GmbH & Co. KG, Germany). This washing process was repeated for a total of 10 cycles until the absorption spectrum of the flow-through no longer changed. At the end, the UV-Visible absorption spectrum of the PpcAK9CK22C cytochrome nanowires was recorded.

### Cytochrome nanowires characterization

### Polyacrylamide gel electrophoresis

The PpcAK9CK22C protein and the 10-µL aliquots from the coupling reaction at different time points were analyzed by Tricine-SDS-PAGE (12.5% polyacrylamide). The electrophoresis was performed on 91.2 pmol of monomer protein, both in the presence and absence of the reducing agent β-mercaptoethanol, at 120 V for 1.5 h. Two identical gels were prepared. One gel was stained with BlueSafe solution to visualize proteins, while the second gel was stained for the peroxidase activity of the heme cofactors, as described by Thomas et al. (1976). Alternatively, the gel was first stained for heme presence and then with BlueSafe to detect total protein. The densitometric quantification of the protein bands on the BlueSafe-stained gel was conducted using the ImageJ software (Schneider et al. 2012; Schindelin et al. 2015).

### Reduction and reoxidation assay

The cytochrome nanowires were subjected to a reduction-reoxidation cycle to assess their electron transfer capacity. A sample with a monomer concentration of 1.4 µM in 50 mM KPB, pH 5.7, 50 mM NaCl was reduced with stepwise additions of solid sodium dithionite, until an excess of the reducing agent was observed on the UV-Visible absorption spectrum. Subsequent reoxidation was then carried out with stepwise additions of 200 µM potassium ferricyanide until the spectrum reverted to its fully oxidized state.

### Synchrotron radiation circular dichroism

Prior to Synchrotron Radiation Circular Dichroism (SRCD) analysis, the PpcA protein and cytochrome nanowires underwent buffer exchange. The samples were dialyzed using a Slide-A-Lyzer™ MINI Dialysis Unit 3.5 kDa (Thermo Scientific™, Thermo Fisher Scientific Inc, USA) to replace NaCl with NaF. The dialysis was carried out for 2 × 1.5 h at room temperature against 100 mL of 50 mM KPB, pH 5.7, 50 mM NaF. The PpcAK9CK22C protein variant was treated with DTT and buffer exchanged as for the coupling reaction. The concentrations of PpcA (2.1 mg/mL), PpcAK9CK22C (2.1 mg/mL), and the cytochrome nanowires (1.0 mg/mL) were estimated based on their absorbance at 205 nm, as described by Anthis and Clore (2013).

SRCD spectra were recorded on the AU-CD beam line at the ASTRID2 synchrotron radiation source (ISA, Aarhus University, Denmark). Measurements were conducted using a quartz cell with a nominal pathlength of 0.01 cm (Quartz Glass High Performance, Hellma Analytics, Germany). The actual exact pathlength of the cell was determined to be 0.01008 cm via an interference technique (Hoffmann et al. 2016). Spectra were recorded in triplicate from 170 nm to 350 nm in 1 nm steps, with a dwell time of 2 s per step. To investigate the thermal stability of the proteins, temperature ramps were performed from 5 °C to 87 °C in 5 °C increments. At each temperature point, a full spectrum was acquired in triplicate. To assess for irreversible changes, spectra were also acquired at 24 °C before and after the temperature ramps. Baseline corrections were performed using the spectra of the flow-through buffer from the final dialysis step during sample preparation.

The melting temperature and enthalpy change of the unfolding transition of the PpcAK9CK22C protein were determined by assuming a two-state thermal denaturation model and using a nonlinear least-squares fit procedure using Eq. 1,

1 $$\:CD=\frac{\left({\alpha\:}_{N}+{\beta\:}_{N}\times\:T\right)+\left({\alpha\:}_{D}+{\beta\:}_{D}\times\:T\right){e}^{-\frac{{{\Delta\:}H}_{m}\left(1-\frac{1}{{T}_{m}}\right)}{RT}}}{1+{e}^{-\frac{{{\Delta\:}H}_{m}\left(1-\frac{1}{{T}_{m}}\right)}{RT}}}$$

where Δ*H*_*m*_ is the enthalpy change of the unfolding transition (cal/mol), *T*_*m*_ is the melting temperature (K), *T* is the temperature (K), *R* is the universal gas constant (1.987 cal·K^− 1^·mol^− 1^), and *𝛼*_*N*_, *β*_*N*_, *𝛼*_*D*_, and *β*_*D*_ are the intercepts and slopes of the pre- and post-transition baselines, respectively.

### Atomic force microscopy

Protein samples of PpcA, PpcAK9CK22C, and cytochrome nanowires (100 nM in 50 mM KPB, pH 5.7, 50 mM NaCl) were applied to a freshly cleaved muscovite mica grade V1 (Ted Pella, Inc., USA) and incubated for 10 min at room temperature. The measurements were performed in liquid medium with an Asylum Research MFP-3D Standalone atomic force microscope (Oxford Instruments, UK), operated in oscillatory mode with commercially available silicon cantilevers (Olympus AC240TS, f_0_ = 70 kHz; k = 2 N/m, Olympus Corporation, Japan) at room temperature and atmospheric pressure. Images were processed with the Gwyddion program (Nečas and Klapetek 2012) and planefitted to correct for background slope errors.

## Results and discussion

### Production and purification of the PpcAK9CK22C variant

**Fig. 1:**
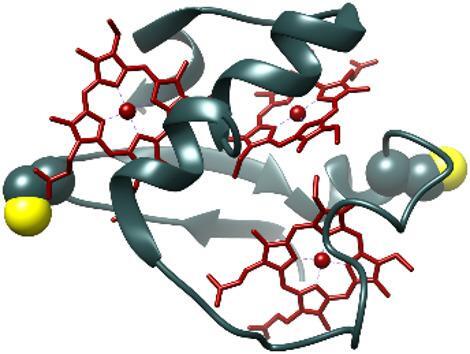
Three-dimensional model of the *Geobacter sulfurreducens* PpcAK9CK22C protein, derived from PDB entry ID 2MZ9. Heme cofactors are shown in dark red. The lysine residues were substituted with cysteine residues, shown as spheres with sulfur atoms highlighted in yellow

To create linear protein polymers that mimic the extracellular nanowires found in *Geobacter* bacteria, the PpcAK9CK22C protein variant was engineered. Lysine residues 9 and 22 were chosen for point mutations because their solvent accessibility and their location facilitate the proper alignment of heme cofactors in the semisynthetic cytochrome-based filaments (Fig. 1).

**Fig. 2:**
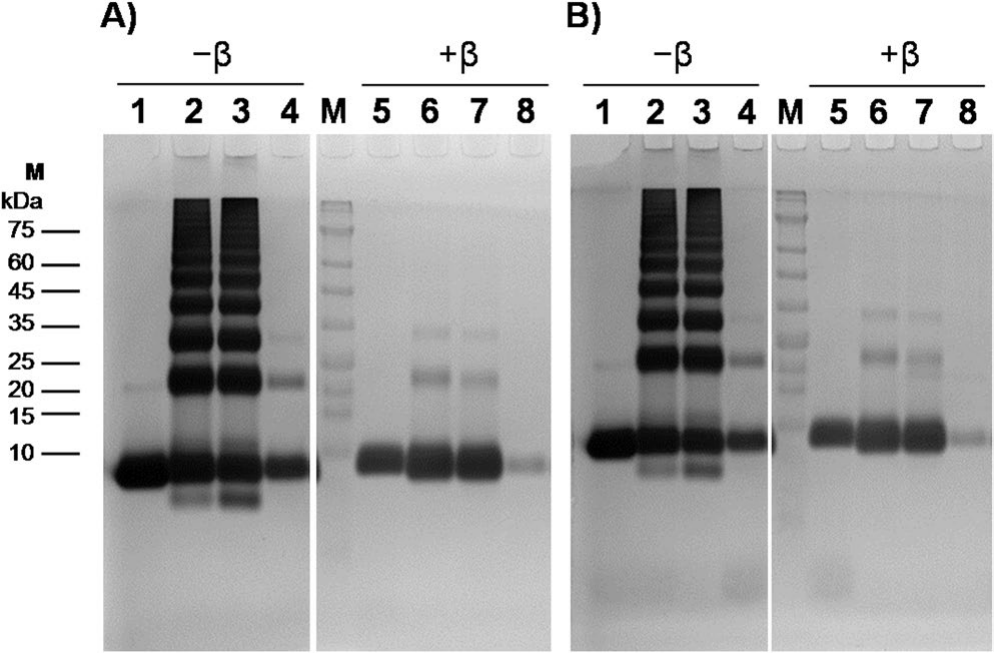
Tricine-SDS-PAGE analysis of PpcAK9CK22C final fractions (12.5% polyacrylamide gel). Lanes + β contain samples with β-mercaptoethanol (reducing conditions), while lanes labeled − β have samples without β-mercaptoethanol (non-reducing conditions). The gel was stained in two steps: first with heme staining (**A**) and then with BlueSafe for total protein detection (**B**). Lanes 1 and 5 – Wild-type PpcA; lanes 2 and 6 – Fraction 1; lanes 3 and 7 – Fraction 2; lanes 4 and 8 – Fraction 3. M – NZYColour Protein Marker I

The recombinant PpcAK9CK22C protein was overexpressed and then purified in two chromatographic steps. The final protein fractions were pooled based on their purity. Analysis through Tricine-SDS-PAGE under reducing conditions (+β lanes, Fig. 2) revealed a single major band with an apparent molecular mass similar to the wild-type PpcA (9.6 kDa). Residual contaminants were visible in fractions 2 and 3 (which appear slightly below the 20 kDa band). Additionally, light bands corresponding to dimers and trimers were also observed. Under non-reducing conditions (−β lanes), the PpcAK9CK22C protein bands exhibited a stepwise increase of ~ 10 kDa, forming a laddering pattern from the monomer band to the top of the gel. This effect was caused by the polymerization of PpcAK9CK22C protein molecules via disulfide bridges, which formed through the oxidation of the newly introduced cysteine residues.

**Fig. 3:**
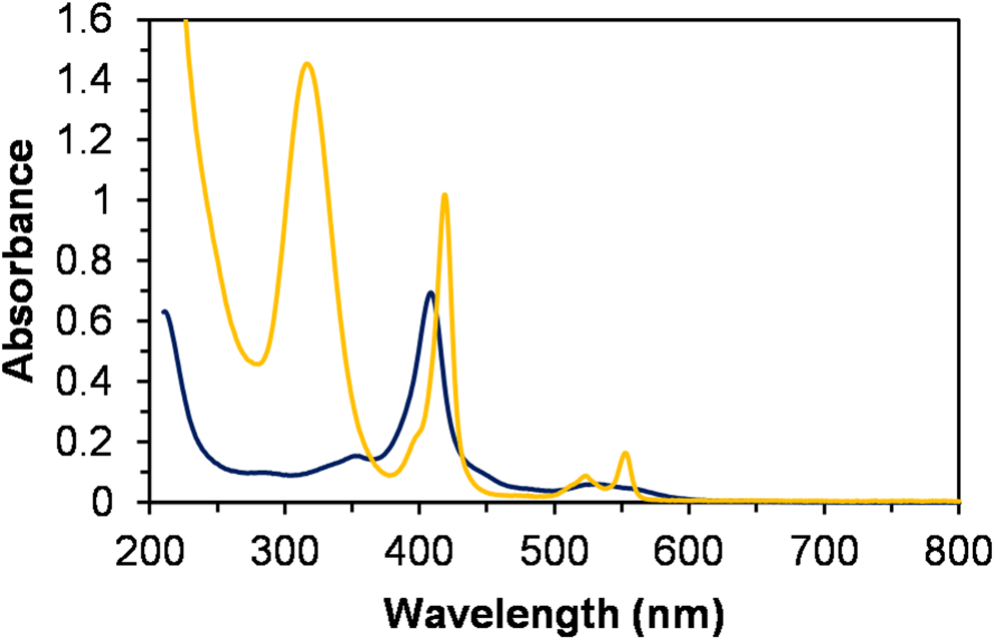
UV-Visible spectra of PpcAK9CK22C protein in the oxidized (blue line) and reduced (yellow line) states

Representative UV-Visible spectra of the oxidized (as-isolated) and reduced forms of PpcAK9CK22C are presented in Fig. 3. The spectrum of the oxidized form is characterized by four absorption bands from the porphyrin π → π* transitions. These included the δ band at 353 nm, the intense Soret (or γ) band at 408 nm, and the two weaker β and α bands at 530 nm and ~ 560 nm, respectively (Meyer and Kamen 1982; Andersson et al. 1983; Moore and Pettigrew 1990; Liu et al. 2014a). In the reduced form, these features sharpened and shifted. The Soret band exhibited a red shift to 418 nm, while both β and α bands displayed blue shifts, to 523 nm and 552 nm, respectively. The intense absorbance observed below 380 nm was attributed to an excess of unreacted sodium dithionite. The overall spectral pattern was typical of low-spin hexacoordinated hemes (Moore and Pettigrew 1990; Inoue et al. 2010), and was consistent with previously reported data for wild-type PpcA (Londer et al. 2002; Fernandes et al. 2008).

The heme-to-protein ratios (Abs_408 nm_/Abs_210 nm_) were calculated from the oxidized spectra. The consistent value of approximately 1 across all fractions confirmed that the PpcAK9CK22C protein was produced as a fully mature protein, successfully incorporating all three heme cofactors (Londer et al. 2002; Fernandes et al. 2008). This result demonstrated that the introduction of the cysteine residues did not disrupt heme binding, despite the relatively proximity of both residues. However, the final yield of purified protein was 0.58 mg per liter of culture, which was 5–10 times lower than that of the wild-type PpcA. (Londer et al. 2002). This reduction may reflect decreased expression and/or impaired folding, cofactor incorporation, or maturation efficiency of the variant, potentially associated with the introduced mutations.

### Characterization of cytochrome nanowires

**Fig. 4:**
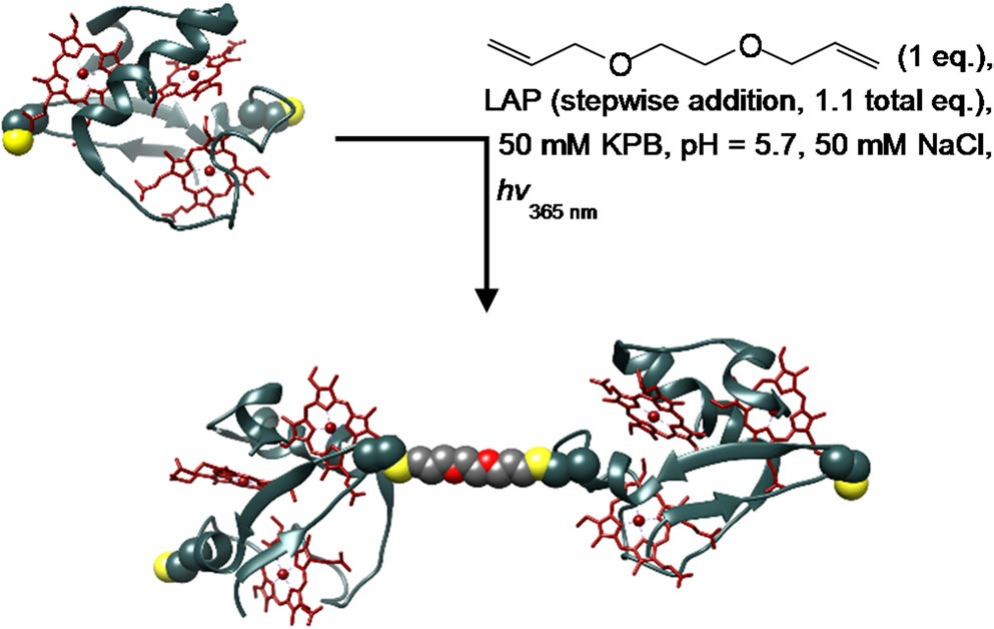
Synthetic approach for cytochrome nanowires production. The diagram outlines the synthesis of cytochrome nanowires via a thiol-ene coupling reaction between the PpcAK9CK22C protein and the 1,2-bis(allyloxy)ethane linker. For clarity, the reaction is only presented at one of the two l-cysteine residues inserted into the protein, and hydrogen atoms are omitted. Heme cofactors are shown in dark red, while the sulfur atoms of the cysteine residues, and the oxygen and the carbon atoms of the linker are highlighted in yellow, red, and gray, respectively. LAP stands for lithium phenyl-2,4,6-trimethylbenzoylphosphinate photoinitiator

Cytochromes nanowires were synthesized through a thiol-ene coupling reaction between the sulfhydryl groups of the PpcAK9CK22C protein and the double bonds of the 1,2-bis(allyloxy)ethane linker. The methodology for this synthetic approach is outlined in Fig. 4.

**Fig. 5:**
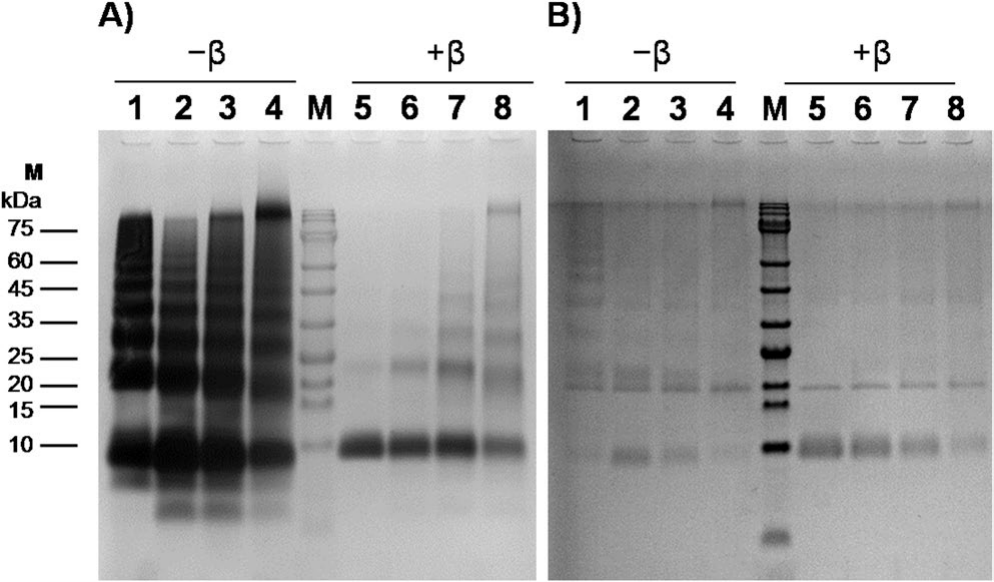
Tricine-SDS-PAGE analysis of the PpcAK9CK22C polymerization reaction (12.5% polyacrylamide gels). Samples in lanes labeled + β were run under reducing conditions with β-mercaptoethanol, and − β under non-reducing conditions. **(A)** Gel stained for heme cofactor. **(B)** Gel stained with BlueSafe for total protein visualization. Lanes 1 and 5 – PpcAK9CK22C pure fraction 2 (control at 0 min); lanes 2 and 6 – Reaction mixture after 30 min; lanes 3 and 7 – Reaction mixture after 60 min; lanes 4 and 8 – Reaction mixture after 120 min. M – NZYColour Protein Marker I

The progress of the coupling reaction was monitored by Tricine-SDS-PAGE (Fig. 5). Analysis of the BlueSafe-stained gel revealed that the initial treatment with 10 mM DTT increased the amount of monomeric PpcAK9CK22C by reducing the existing disulfide bridges (lanes 1 and 5). As the reaction time increased, the intensity of the monomeric band decreased, while higher molecular mass bands appeared up to the top of the gel, indicating polymerization of the PpcAK9CK22C molecules.

This behavior was more noticeable in samples analyzed under non-reducing conditions (−β lanes) but was also observed under reducing conditions (+β lanes). This confirmed that thioether bonds were successfully formed during the coupling reaction. After 120 min, the reaction reached a yield of ~ 89%, with 75% of the protein polymerized through thioether bonds and 14% bound via disulfide bridges, as determined by densitometric quantification of the protein bands. It is also worth noting that the intensity of the contaminating protein band (~ 20 kDa, as seen in gels in Fig. 2) remained constant throughout the reaction, confirming that this impurity was not incorporated into the final nanowire product.

**Fig. 6:**
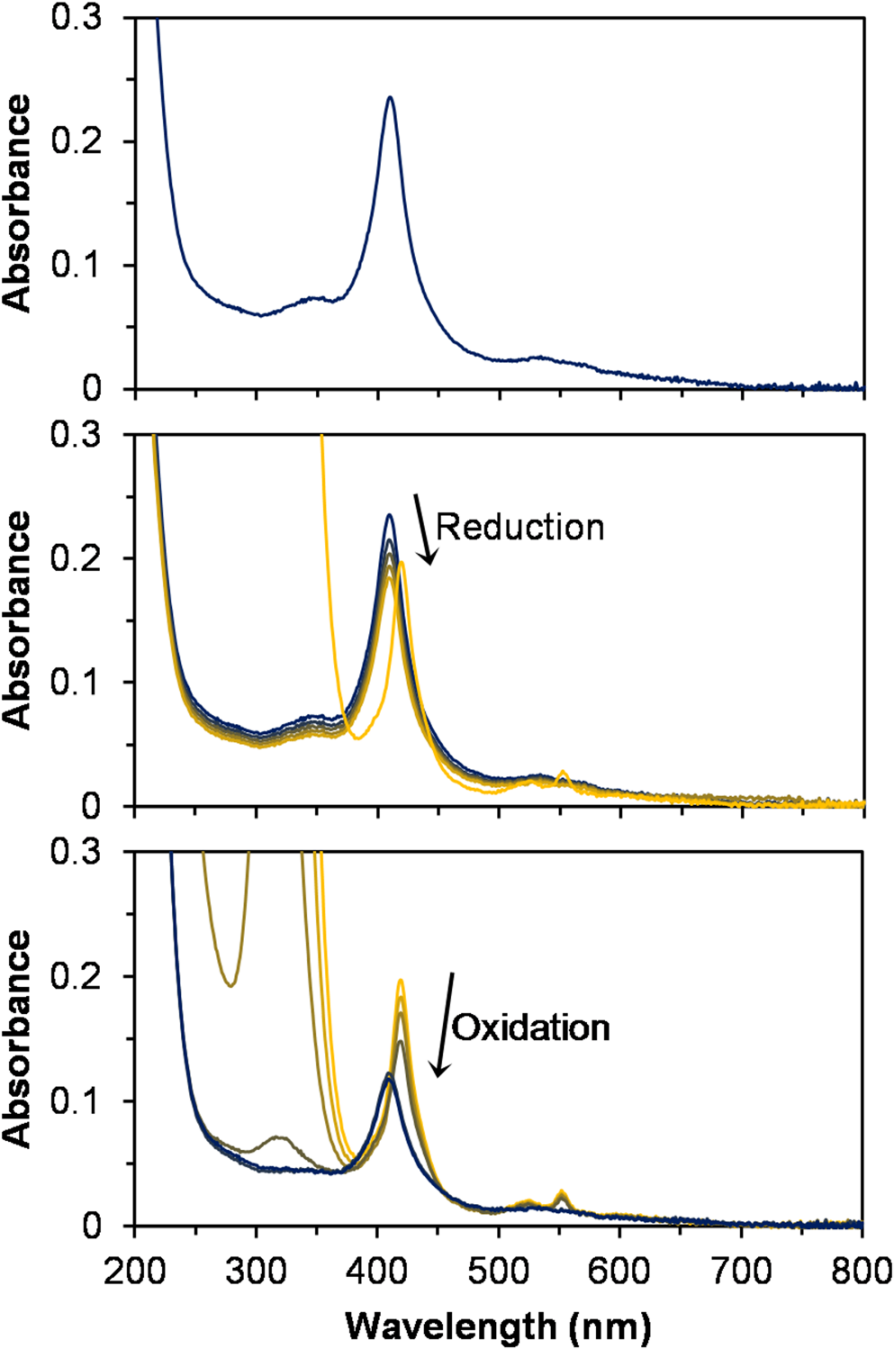
Redox cycle of the cytochrome nanowires, monitored by UV-Visible spectroscopy. The top panel shows the spectrum of the PpcAK9CK22C coupling reaction product, while the stepwise reduction and subsequent reoxidation are presented in the center and bottom panels, respectively

The UV-Visible spectrum of the coupling reaction product (Fig. 6, top panel) presented absorption bands similar to those of the oxidized precursor protein. However, a subtle increase of the absorbance was observed in the 600–700 nm region, likely due to the formation of high-spin heme groups (Moore and Pettigrew 1990). Moreover, the heme-to-protein ratio decreased to ~ 0.5, indicating that nearly 50% of the heme groups were destabilized during the synthesis of the nanowires. The instability of the heme groups was also observed during a reduction-reoxidation cycle. As shown in the center panel of Fig. 6, the intensity of the Soret band decreased along the reduction process, which contrasts with the behavior of the PpcAK9CK22C protein. Following reoxidation (Fig. 6, bottom panel), the nanowires returned to the oxidized state, but recovering only ~ 52% of the initial intensity of the Soret band.

**Fig. 7:**
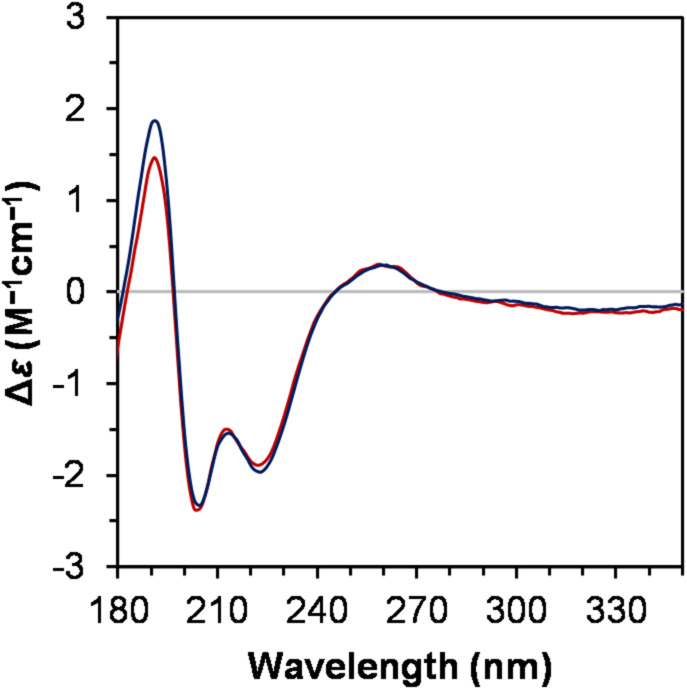
SRCD spectra of wild-type PpcA (red) and PpcAK9CK22C (blue) proteins at 5 °C. The samples contained 2.1 mg/mL of protein in 50 mM KPB, pH 5.7, 50 mM NaF.

The PpcAK9CK22C protein exhibited an SRCD spectrum very similar to that of the PpcA wild-type protein (Fig. 7), with two positive bands at 191 nm and 258–260 nm, and two negative bands at 204–205 nm and 222 nm. Such spectral agreement provides strong evidence that the variant maintained the structural integrity and folding of the wild-type protein, as the high energies and photon flux available with SRCD enable sensitive detection of secondary-structure content and subtle conformational changes that would otherwise be difficult to discern.

**Fig. 8:**
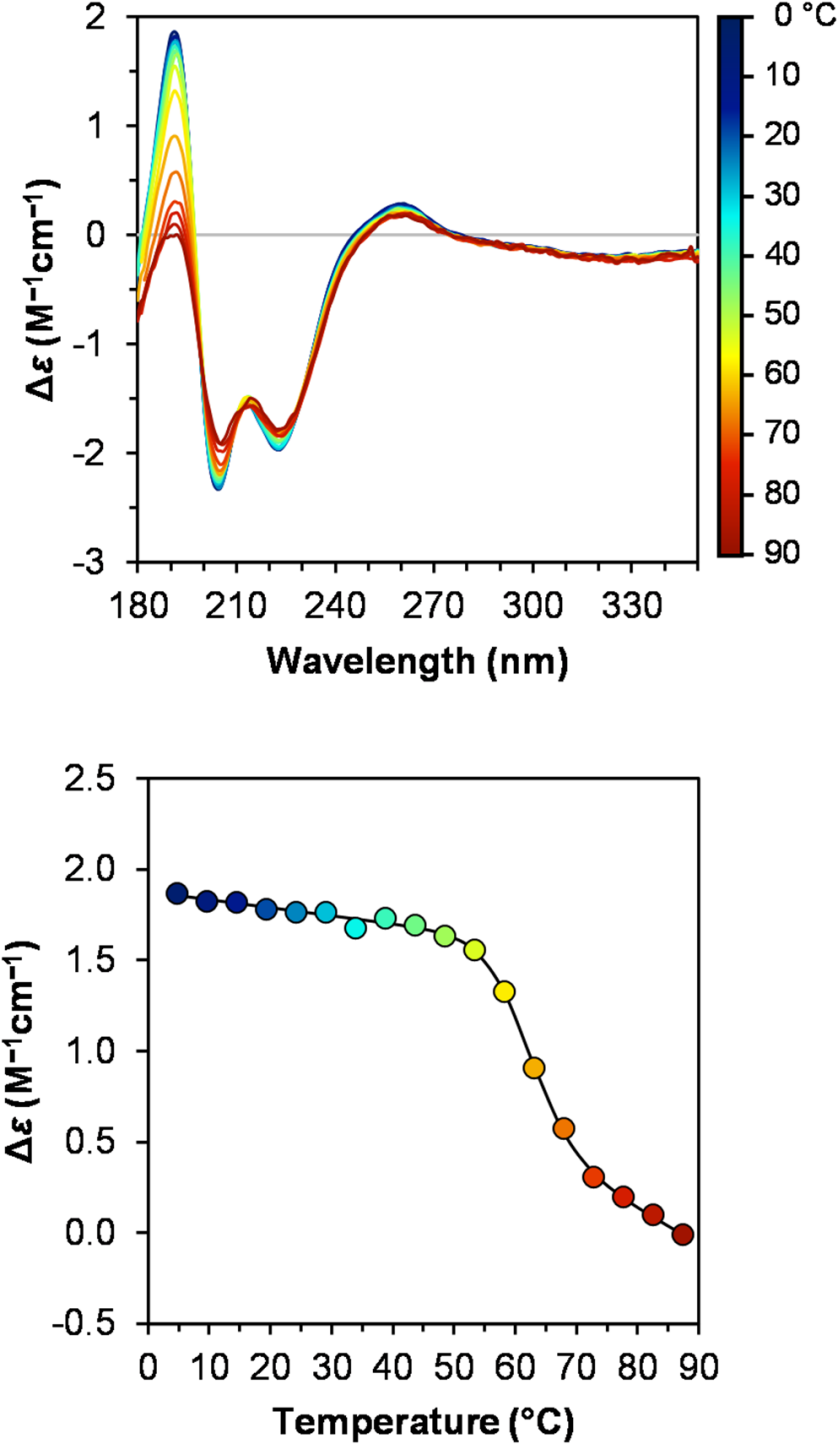
Thermal stability analysis of PpcAK9CK22C protein by SRCD spectroscopy. The top panel shows the temperature ramp between 5 °C and 87 °C, while the melting curve at 191 nm is presented in the bottom panel with the obtained non-linear least-squares fit (solid line). The sample contained 2.1 mg/mL of protein in 50 mM KPB, pH 5.7, 50 mM NaF.

**Fig. 9:**
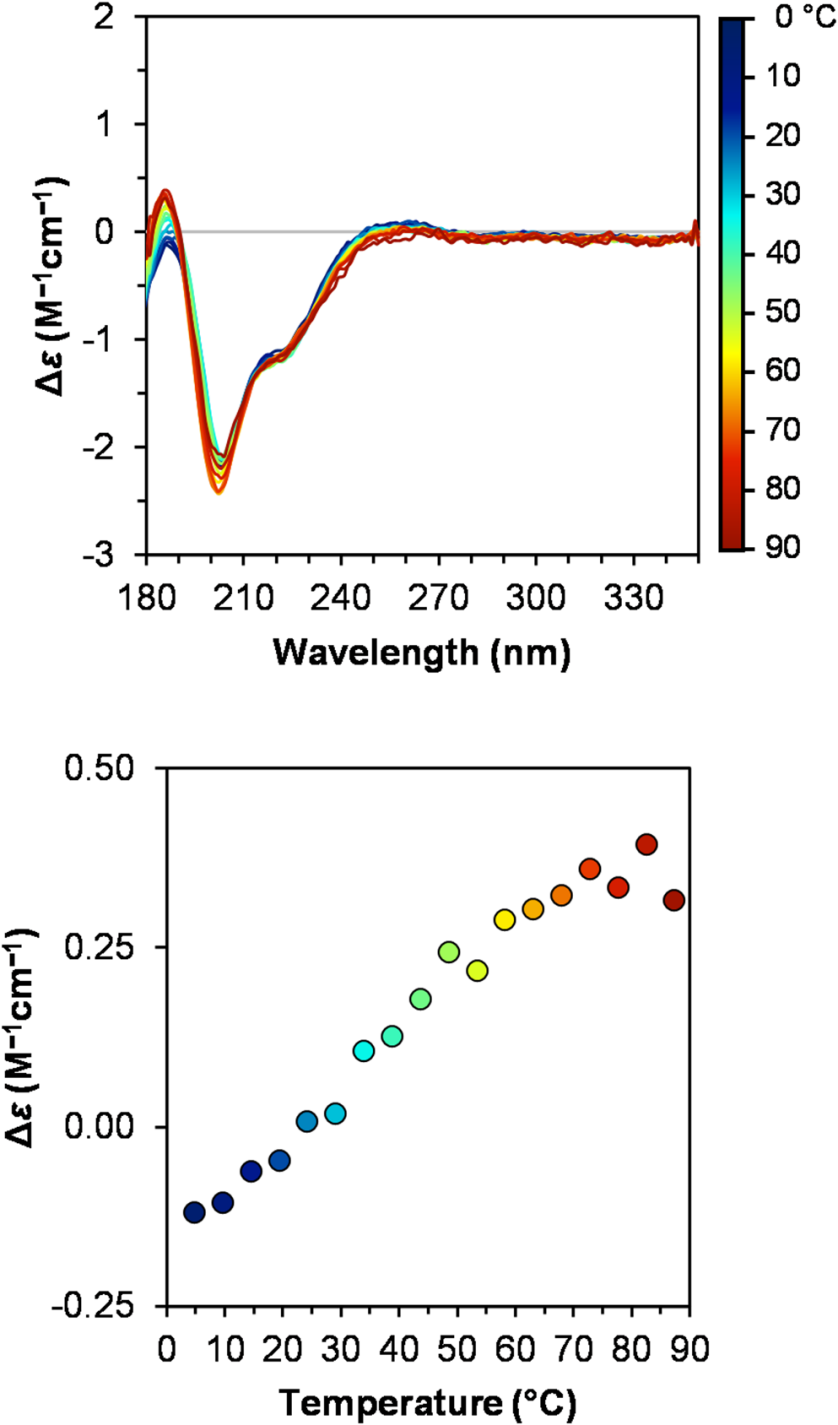
Thermal stability analysis of cytochrome nanowires by SRCD spectroscopy. The top panel shows the temperature ramp between 5 °C and 87 °C, while the melting curve at 186 nm is presented in the bottom panel. The sample contained 1.0 mg/mL of protein in 50 mM KPB, pH 5.7, 50 mM NaF.

The thermal stability and the secondary structure of PpcAK9CK22C protein and cytochrome nanowires were assessed using SRCD spectroscopy (Figs. 8 and 9). For the PpcAK9CK22C protein, a two-state thermal denaturation process was observed with a melting temperature of 62 ± 1 °C and an enthalpy change of 61 ± 2 kcal/mol.

Structurally, PpcA proteins are known to display a significant degree of plasticity, which is undoubtedly related to their specific electron transport function. Secondary structural description obtained from the NMR structure of PpcA (PDB entry ID 2MZ9) using the 2Struc server (Klose et al. 2010) showed the following ranges: 8–37% for helices, 0–15% for β-sheets, and 51–92% for other structures (including turns), due to the fact that secondary structure content could vary depending on the chosen conformer and assignment method. Our SRCD data are consistent with the values cited above, while also showing that for temperatures above 50 °C, there is a gradual loss of helical structure accompanied by a concomitant gain in β-sheet and/or unordered components.

Interestingly, the SRCD spectra of the PpcAK9CK22C nanowires showed striking changes relative to the precursor protein. The positive bands at 191 nm and 260 nm were no longer evident, and the negative band at 222 nm appeared only as a shoulder of the 203 nm band. These spectral changes are consistent with a small reduction in helical content and an increase in β-sheet and unordered structures. Considering the inherent plasticity of PpcA proteins and the structural location of the introduced cysteine residues (see Fig. 4), it is reasonable to envision that the polymerization reaction induces a structural stretching that disrupts regular α-helical elements.

Furthermore, the thermal behavior of the nanowires was markedly different and could not be adequately explained by the same denaturation model applied to the precursor protein. As shown in Fig. 9, only minor spectral changes were observed over the same temperature range tested for the precursor protein. This suggests that the nanowires undergo only small structural rearrangements and maintain their integrity between 5 °C and 87 °C. Moreover, the gradual nature of these minor changes, without a sharp transition, is characteristic of a non-cooperative unfolding process, in which structural elements denature independently rather than in the all-or-none manner typical of cooperative two-state transitions.

**Fig. 10:**
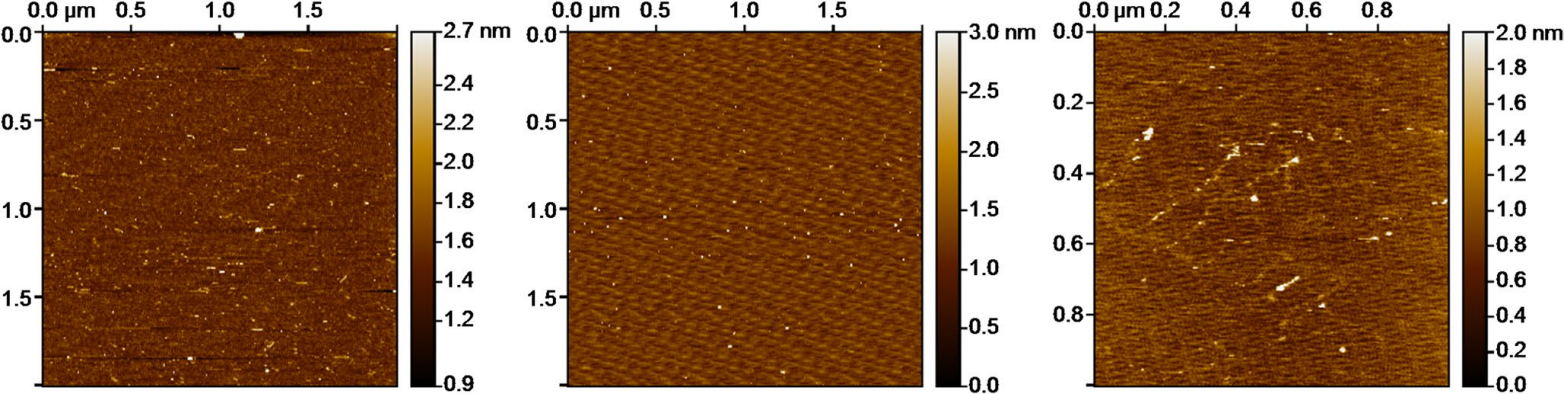
AFM topographic images in liquid medium. Images of wild-type PpcA, PpcAK9CK22C, and cytochrome nanowires are displayed from left to right, respectively.

The synthesized cytochrome nanowires were analyzed by AFM to study their size and morphology in comparison with PpcA and PpcAK9CK22C proteins. The images acquired in liquid medium (Fig. 10) showed that both PpcA and PpcAK9CK22C proteins were visible as small, brighter dots. PpcA was measured to have a width of 20 ± 4 nm and a height of 1.1 ± 0.7 nm (*n* = 100). PpcAK9CK22C had a similar width (18 ± 4 nm) but a slightly greater height of 3 ± 2 nm (*n* = 100), consistent with experimental uncertainty. The measured width values were approximately 5 times larger than the protein’s 4 nm size determined from the NMR structure. This discrepancy could be explained by the tip-sample convolution effect, which caused a lateral broadening artifact (Shen et al. 2017). The observed differences in height, when compared to the NMR structure, may result from conformation distortions caused by protein interactions with the muscovite mica surface (Barinov et al. 2016). Given that PpcA has two additional lysine residues on its surface, its higher degree of distortion compared to PpcAK9CK22C may stem from its ability to establish more interactions with the mica. Imaging of the cytochrome nanowires revealed fibers longer than 300 nm with a width of 20 ± 6 nm (*n* = 12). This finding indicated that each protein wire was likely composed of a single polymer chain of cytochromes.

## Conclusions and future perspectives

To our knowledge, this work reports the very first synthesis of cytochrome nanowires through the direct polymerization of a multiheme *c*-type cytochrome. A mutated variant of the triheme periplasmic protein PpcA was chosen as the building block, with protein coupling achieved through a thiol-ene reaction.

The designed PpcA variant, PpcAK9CK22C, was successfully produced as a fully mature protein. This protein variant showed no significant structural differences from the native protein. It had a defined melting temperature and an enthalpy change for the unfolding transition, suggesting that it underwent a two-state thermal denaturation process.

The cytochrome nanowires were found to have lengths of hundreds of nanometers and a lower heme group content than their precursor protein. The nanowires were partially redox-active, as evidenced by incomplete recovery after reduction. They also exhibited a distinct secondary structure, characterized by fewer α-helices and more β-sheets and other structures, along with improved thermal stability. This enhanced stability strengthens their suitability for potential applications, indicating that the nanowires maintain structural integrity and functional properties under conditions exceeding room temperature. Future work is needed to improve the stability of the heme groups during the coupling reaction and to enhance the recovery yield in redox cycles. Additionally, the SRCD spectra seem extremely adequate to evaluate the protein structure stability, prompting its use in further studies of both wild-type and variant proteins.
